# Kienböck's Disease: A Narrative Review of Pathophysiology, Etiology, and Classification Systems

**DOI:** 10.7759/cureus.98211

**Published:** 2025-12-01

**Authors:** Ruqaiya Al-Habsi

**Affiliations:** 1 Surgery, The Royal London Hospital, London, GBR

**Keywords:** avascular necrosis, avascular necrosis (avn), kienböck disease, kienböck's disease, lunate bone

## Abstract

Kienböck’s disease affects the lunate bone and results in avascular necrosis. It remains a disease that is not widely understood in terms of disease progression and the pathophysiology surrounding it. Despite multiple studies existing about the surgical management options, there is still no consensus about the ideal surgical modality for it. In this narrative review, the aim was to read and summarise existing literature about the pathophysiology, causes, and current classification systems. The search databases used included PubMed, EMBASE, and Google Scholar with search words, including “Kienböck”, “Kienböck disease”, “lunate avascular necrosis”, “Lichtman classification”, “Kienböck classification”, ‘Kienböck litigation”, “avascular necrosis”. The majority of published research focused mainly on the treatment options and history of the disease, with a limited number of papers about the etiology, pathophysiology, and progression.

## Introduction and background

Avascular necrosis of the lunate bone is known as Kienböck’s disease (KD), first explained in 1910 by Robert Kienböck [1-3]. KD occurs due to disturbance to the lunate blood supply, which causes necrosis, structural collapse, degenerative changes to the surrounding carpal joints, and osteoarthritis [1,4]. It mainly affects adults between 20 and 40 years of age and is more common in males, and patients usually have a previous trauma [5]. It is known by multiple names historically, usually in relation to the aetiology, and these include lunatemalacia, aseptic necrosis, osteochondritis, osteitis, and traumatic osteoporosis of the lunate. The main current aetiology thought to be causing KD is aseptic lunate necrosis [6].

A large cross-sectional review of 51,071 patients over an 11-year inclusion period reported a prevalence of 0.27%, with 37% of cases being incidental cases and asymptomatic patients [7]. Another study based on a retrospective review of 150,000 plain radiographs showed a low incidence of KD, with an estimated seven in 100,000 patients, making it an uncommon disease [3].

The exact cascade of events causing KD has not been fully understood. Various theories exist about vascular perfusion, anatomical variations, repeated trauma or microfractures, and systemic disease [8]. Prior to the discovery and development of plain radiographs in 1895 by Wilhelm Roentgen, a German physicist, anatomists relied on dissection of cadavers to analyse carpal bone anatomy and related pathology. These early discoveries showed various congenital abnormalities of the lunate bone, including lunarum partition, hypolunatum, epilunatum, and bipartatum [9]. It has been more than 100 years since KD was first described in literature, and there remains no consensus on the etiology of this disease [10].

The functional prognosis of KD is still unclear; it is known that, as it progresses in severity, it can result in degenerative wrist changes. Prognosis is not usually good with conservative management in adult patients; therefore, surgery is needed in the majority of cases [11]. The choice of surgical treatment varies based on the severity of the disease; however, there is no consensus yet about which surgical options are superior to others.

The aim of this narrative review is to summarise the current understanding of the anatomy, biomechanics, proposed etiological factors, and classification systems of KD, with a brief overview of how these frameworks inform treatment principles.

## Review

Methodology 

This narrative review was conducted using PubMed, EMBASE, and Google Scholar up to October 2025. Search terms included ‘Kienböck’, ‘Kienböck disease’, ‘lunate avascular necrosis', ‘Lichtman classification’, ‘Kienböck classification’, ‘Kienböck litigation’, and ‘avascular necrosis’. Human studies published in English involving all age groups were included, while animal studies and non-clinical experimental articles were excluded. As this is a narrative review, no formal risk-of-bias assessment was performed.

Relevant anatomy

The lunate bone, which can be considered the nucleus of the wrist joint [12], is half-moon shaped when viewed sideways. Proximally, it is convex, and distally it has a concave morphology. It articulates with the radius, and the triangular fibrocartilage complex (TFCC) proximally and distally articulates with the head of the capitate and, in some variations, with the hamate bone [11].

Antuña-Zapico classified lunate morphology into five types and also highlighted an existing relationship between lunate morphology and its strength. Type I has an angle greater than 130°, type II has an angle of 100°, and type III has two facets proximally: one for radius articulation and the other articulating with the TFCC [13].

The carpal bones are supplied by five main blood vessels: the ulnar, radial, anterior interosseous arteries, the accessory ulnar recurrent artery, and the deep palmar arch. These vessels create a volar and dorsal network to supply the carpal bones. Each vessel is further divided into transverse arches where nutrient arteries branch off to supply the bones [14].

The lunate receives blood supply through volar and dorsal entry points. The dorsal pole receives supply from the radiocarpal arch vessels. Volarly, the lunate is supplied by the palmar plexus branches in addition to a direct supply from radial, anterior interosseous, and ulnar branches [13].

Lunate biomechanics 

Wrist joint movements, whether flexion or extension, also need flexion and extension of both the proximal and distal carpal rows. During wrist movements, the articulation and relationship of the lunate, capitate, and radius vary. When the wrist is held in ulnar deviation, the lunate is fully covered by the radius bone, and, in turn, it partially covers the capitate. When the wrist is held in radial deviation, the lunate is partially covered by the radial fossa and supported by the TFCC. These articulations and changes in relationships between these bones are important to understand load distribution in the wrist joint and carpal bones [15].

There is an unequal distribution of the load transmitted through the carpal bones between the TFCC, the scaphoid, and the lunate bone. The distribution is essential for the maintenance of wrist position and ulnar deviation. In early KD stages, this weight distribution is not affected. As the disease progresses, the lunate collapses and scaphoid bone position changes, becoming more horizontal, reducing the stress on the lunate fossa, and increasing within the scaphoid fossa. This change in load distribution can result from advanced destruction of the lunate bone [16].

Pathophysiology, risk factors, and aetiology 

Necrosis of bones, known as osteonecrosis, can be a result of various factors, resulting in avascular and necrotic bones. Repetitive traumatic injuries and insufficient collateral circulation can render carpal bones vulnerable to osteonecrosis, which typically can involve the scaphoid bone, capitate, and lunate bones. In KD, even when a traumatic event or actual cause is unknown, the final event resulting in the disease is usually the same: an underlying vascular compromise resulting in critical ischaemia [17].

The duration of disease progression varies from one patient to another, and it can take months to years between the initial symptoms and the development of arthritis. In some literature, it is noted that worsening radiological stages may not result in equally worsening clinical effects [5]. Osteonecrosis of the lunate bone is the main pathology in KD. As the lunate is a keystone to the normal functioning and anatomy of the wrist, lunate collapse and reactive synovitis can affect surrounding carpal bone anatomy and result in degenerative arthritis [18].

In 2025, Koo et al. [19] published a study looking at the radiologic prevalence of KD in Korea. They performed a retrospective study on adult patients who underwent hand or wrist plain radiographs, CT scanning, and magnetic resonance imaging (MRI) between January 2010 to December 2020. Their study yielded a total of 61,903 radiological reports, which met their study criteria. It was noted that KD was prevalent in 0.17% with a higher incidence in females (0.17%) in comparison to males (0.16%) [19]. Of all KD cases, there is an estimated incidence of 3-7.3% of bilateral KD [2].

Negative ulnar variance has long been proposed as a mechanical risk factor for KD development. Some studies, such as that by Wassef et al. [10], demonstrate a correlation between lower radial inclination angle and later KD stages. This anatomical pattern is widely discussed as a predisposing factor for both disease development and progression. However, evidence remains mixed, and its role should be interpreted with caution [10,20].

Chung et al. [20] conducted a meta-analysis to evaluate whether negative ulnar variance is in fact a risk factor for developing KD. They reviewed studies published between 1966 and December 2000, and only three main studies met their inclusion criteria. One study showed a significant link between the two, while overall the pooled findings demonstrated only a trend towards significance rather than definitive proof [20].

The etiology of KD can be divided into categories such as mechanical factors, vascular factors, and others. Mechanical factors can be further subdivided by extrinsic factors, such as ulnar variance, radial inclination, and type of load. Table 1 shows possible mechanical factors as a cause for KD [21]. Although multiple theories have been published about these possible etiologies, an actual understanding of the role of each of those has not been clearly defined [22].

**Table 1:** Proposed mechanical etiologies of Kienböck’s disease (KD) Source: Lluch et al. [21] and Salva-Coll et al. [22]

Extrinsic mechanical factors	Intrinsic mechanical factors
Repetitive load trauma or manual laborers can present with necrosis caused by repetitive mechanical stress on the lunate bone.	Negative ulnar variance has been reported to increase risk factors, but recent reviews and meta-analyses show a possible association, and the literature on this is limited.
Acute or chronic trauma to the lunate bone can result in injury to the bony anatomy of the surrounding vasculature, eventually resulting in insufficient blood supply and necrosis.	Positive ulnar variance can result in worsening of load distribution within the wrist joint, leading to lunate stress.
Occupational vibration exposure can result in repetitive microtrauma to the wrist and lunate bone, but evidence that this can ultimately result in KD is weak.	Variations in distal radius anatomy and how it articulates with the lunate can increase load and reduce articular surface area between the two bones.

Several studies have proposed vascular risk factors that can result in KD, varying from synovitis to circulation impairment. The lunate receives blood supply from both dorsal and volar entry sites, and variations in this can result in vascular supply impairment. The resultant impaired circulation or venous outflow can, in turn, result in venous stasis, which can increase intraosseous pressure and contribute to the development of or worsening of existing KD [21].

Some studies suggest systemic diseases, such as those needing repeated corticosteroid use, sickle cell anemia, and steroid-induced hypercoagulability, can often be associated with KD but have an unclear role in this. There is no definitive evidence that KD has a familial predisposition, but some studies have shown familial clustering [22].

In addition to the above etiologies, Balassiano et al. [23] published a case report in 2024, identifying a link between KD and ulcerative colitis (UC), an inflammatory bowel disease, which is known to cause chronic inflammation and ulcerations in the linings of the colon and rectum. Patients with UC have associated manifestations, such as chronic peripheral joint inflammation, weakening wrist joint structure, ultimately resulting in KK. This is further worsened by the joint toxicity caused by chronic steroid and fluoroquinolone use in UC patients [23].

Further connection has been noted in patients with cerebral palsy (CP), who have a higher incidence of the disease. This is likely due to habitual wrist flexion, spasticity, and resulting injury to volar vessels. Some studies reported a 2-10% incidence of KD in CP patients [9]. The most recent causative pathology theory is that repetitive stress on the lunate bone results in increased intra-osseous pressure, affecting arterial blood supply and resulting in a hypoperfused lunate. This can result in ischaemia, oedema, and marrow fat necrosis [22].

Clinical presentation 

The stereotypical patient presenting with KD is a manual laborer with activity-related signs and symptoms affecting the dominant side [9]. Patients with KD often present with centralised wrist pain, reduction in grip strength, and a reduced range of movement. This can be unilateral or bilateral [19]. Due to this, it is difficult for KD to be diagnosed based only on clinical findings and requires further investigations.

Recent literature publications have shown that KD not only affects middle-aged, manual worker males but actually has two additional types of individuals it affects; these are the following: older females with a positive ulnar variance and younger teenage “teenbock” patients who have self-limiting KD. Even though KD affects males more than females, the latter actually have a faster disease progression radiographically. This can be due to a multitude of reasons, including age-related osteoporosis, which is usually more common in females than males [24].

Patients presenting with bilateral KD do not necessarily have any synchrony in their symptoms. There is still very limited research and understanding about bilateral KD, and most studies focusing on unilateral KD symptoms vary from generalised wrist pain, most commonly central, wrist swelling dorsally, and reduced range of motion [9]. On examination of some patients, tenderness may be present between the extensor digitorum tendon and the extensor carpi radialis tendons. With time, stiffness worsens and begins to affect wrist range of movement, eventually reducing power [16].

In many instances, KD can be asymptomatic and is often an incidental finding, with some studies estimating an asymptomatic prevalence of KD between 1% in African populations and 2% in Japanese populations. In America, a large retrospective study took part, analysing more than 51,000 patients who had wrist radiographs taken for reasons other than KD, and showed an overall incidence of KD to be 0.27%, out of which 0.17% showed some symptoms of KD and 0.10% were asymptomatic [9].

Diagnosis, staging, and classification 

Imaging is central to the diagnosis and staging of KD and directly guides management decisions. Plain radiographs are typically the first-line investigation and allow recognition of sclerosis, collapse, and carpal alignment changes, but they cannot fully assess internal lunate structure.

High-resolution computed tomography (CT) is particularly useful for evaluating cortical integrity and subtle fragmentation of the lunate, which is important when planning surgical options [4,22]. Magnetic resonance imaging (MRI) provides complementary information on bone marrow oedema, vascularity, and cartilage condition and is widely used for early detection and MRI-based staging systems. 

Wrist arthroscopy offers direct visualisation of the chondral surfaces and central column and can refine staging and guide treatment; however, it is invasive and is generally considered an adjunctive tool rather than a routine first-line diagnostic modality [25].

The Lichtman Classification

A commonly used classification system is known as the Lichtman classification. It stages the disease into four different stages, ranging from stage I, which indicates normal radiographs, to stage IV showing pancarpal arthritis [1]. Table 2 shows the Lichtman classification, in addition to plain radiograph and MRI findings for each stage.

**Table 2:** Lichtman classification and corresponding radiological features Source: Beyyato et al. [1] and Daly et al. [9]

Disease stage	Radiograph findings	MRI findings	
		T1	T2
I	No abnormalities on X-rays	Low	Variable
II	Lunate sclerosis without any collapse. Preservation of carpal bone alignment	Low	Variable. Scans may show early signs of oedema. It can progress to a low signal when sclerosis or fibrosis is dominant
IIIA	Lunate collapse with preservation of carpal bone alignment. Radioscaphoid angle <60º	Low	Variable. Can often be mixed with oedema zones adjacent to areas of necrosis or sclerosis
IIIB	Lunate collapse with fixed scaphoid bone in flexion/rotation position. The radioscaphoid angle is >60º with fixed carpal bone malalignment	Low	Often low due to chronic sclerosis or fibrosis. In some cases can be patchy if there is active oedema
IIIC	Lunate bone with fractures on coronal plane (different from IIIB malalignment)	Low	Variable. Oedema surrounds the fracture line and chronic segments are low
IV	Pancarpal arthritis (degenerative changes beyond the lunate bone)	Low	Low (due to chronic necrosis or sclerosis). Less oedema is noted in these scans

This classification system was initially published in 1977 and was widely used for over 50 years. It focuses on plain radiographs of the wrist joint, which help show the progressive disease stages. MRI allows the further subdivision of stage III into IIIA and IIIB based on the presence of oedema and hypointensity in T2-weighted scans [26].

The Bain and Begg Classification

A second classification, known as the Bain and Begg classification, initially published in 2006, is based on arthroscopic findings [27]. It assesses articular surfaces and classifies based on articular changes, from grade zero to grade four, while looking at the number of non-functional articular surfaces [18]. Table 3 summarises the Bain classification method for KD. When using this classification, non-functional articular surfaces are defined as those exhibiting one of the following during arthroscopic investigation: fissuring, extensive fibrillation, floating articular surface, extensive articular loss, or a fracture [27].

**Table 3:** Bain and Begg classification and corresponding arthroscopic features Source: Bain et al. [18] and Lichtman et al. [7]

Bain grade	Arthroscopic changes in the central column	Number of non-functional articular surfaces	Corresponding Lichtman stage(s)
0	All articular surfaces are functionally structured with cartilage firm on probing	0	I (early)
I	Most commonly, the proximal lunate articular surface is affected. Opposing surface remains intact	1	II (or early IIA)
IIA	Two functional articular surfaces: the proximal lunate and lunate facet of the radius. The distal lunate or distal cartilage may be borderline but remain firm and stable on probing	0-1	II-IIIA
IIB	Two non-functional articular surfaces: the proximal and distal articular surfaces of the lunate. The radial facet remains intact and the capitate head is congruent	2	IIIA (or IIIX if lunate fracture present)
III	Three non-functional surfaces: the lunate facet of the radius, the proximal, and the distal surfaces of the lunate. The capitate remains intact and unaffected	3	IIIB-IIIC
IV	All four articular surfaces are non-functional (the radial facet, both lunate articular surfaces, and the capitate bone)	4	IV

The Bain-Lichtman Classification 

Studies on MRI-based classifications, different from the Litchman classification, have also been published and involve assessment of lunate cortical integrity, bone marrow edema, and dorsal subluxation of the scaphoid. Table 4 shows the MRI-based classification of KD. 

**Table 4:** MRI-based classification of Kienböck’s disease Source: Joo-Yul et al. [28]

MRI-based classification stage	Core MRI marrow and cortex signals	Carpal bone alignment and collapse	Cartilage condition and arthrosis
Stage I	Patchy bone marrow oedema	No loss of cortical integrity with scaphoid alignment preserved	No degenerative changes visible
Stage II	Oedema may persist. Loss of cortical integrity with focal cortical breach or subchondral signal changes	No gross lunate collapse with continued preservation of carpal alignment	No arthrosis
Stage IIIA	Mixed signals with subchondral changes. Evidence of early structural compromise	Lunate collapses but no dorsal scaphoid subluxation seen	Minimal changes or no changes
Stage IIIB	Unchanged from IIIA (Mixed signals with subchondral changes. Evidence of early structural compromise)	Lunate collapse with dorsal scaphoid subluxation	Minimal changes to cartilage with no or early arthrosis
Stage IV	Radioscaphoid, radiolunate, radiocapitate and/or capitoscaphoid degenerative arthritis with variable marrow signal	Progressive carpal malalignment	Pancarpal degenerative changes

Treatment modalities

The following section provides a brief overview of treatment principles, primarily to demonstrate how classification influences management decisions rather than to offer an exhaustive review of surgical techniques.

KD management significantly varies depending on the state of the lunate [29], and there is no consensus on the ideal management for the various KD stages [30]. One of the most important factors contributing to the choice of treatment and offering an insight into prognosis is the staging. Patients presenting in the early stages are often offered conservative management as opposed to those with more advanced stages, who are offered surgical management ranging from shortening osteotomy of the radius and arthrodesis to arthroplasty [19].

In order to appropriately manage a necrotic bone, the main approach aims to revascularise it. This can involve direct revascularization techniques, such as vascularized bone grafting, which helps in both re-establishing blood flow, as well as provision of mechanical support to the affected lunate bone. Commonly, the donor site is from the distal radius, as it contains an adequate blood supply for this to be effective. Some studies even show success rates up to 85% for patients undergoing these procedures, based on clinical reviews 31 months postoperatively, on average [6].

The choice of treatment modality varies depending on the Lichtman and Bain classification [30]. It is possible to divide the management options into four main groups: joint lunate unloading/joint leveling, core decompression, lunate revascularisation, and salvage surgeries [8]. There are various surgical procedures that have been suggested for the management of the disease. However, existing literature still shows we have not established which methods are more surgically superior [5]. The choice of management depends on patient age, severity of the disease and staging, and whether the lunate bone remains viable or not. The options include lunate decompression for early disease stages to excision and exchange with a prosthetic in more severe cases [26].

The treatment of KD varies significantly based on the Lichtman stage of the disease. Although there is no consensus on the most appropriate surgical or non-surgical modality for each stage, the general management for Lichtman stage I is immobilisation. Stages II and IIIA can often be treated surgically with radial or capitate shortening osteotomy, whereas stage IIIB can undergo more complex procedures such as proximal row carpectomy (PRC), scaphotrapezoidtrapezoid (STT), or scaphocapitate (SC) arthrodesis. Stage IV can be managed with arthrodesis of the wrist or arthroplasty if it is unsuccessful [7].

At KD's early stages, patients can be managed conservatively, initially managed with splinting or application of short arm casts for a minimum duration of three months. Usually, at early stages, surgery is not offered unless the patient remains symptomatic despite these measurements. In Lichtman stage II, the main goals for treatment are to unload the lunate, decompress it, and revascularize. In stage II treatment varies depending on ulnar variance, with radial shortening osteotomy offered to those with a negative ulnar variance, and remains the most commonly used procedure for joint-levelling [30].

Limited wrist fusion generally involves STT and SC fusion. The general indication for this treatment modality is advanced stages of Lichtman classification: stages IIIA, IIIB, and IIIC [31]. PRC is generally indicated for Lichtman disease stages IIIB, IIIC, and IV. PRC is not usually recommended for patients younger than 35 years of age or those who need to use manual strength due to the risk of radiocapitate joint arthrosis and reduction in grip strength [31]. This technique is dated but remains an effective salvage procedure if the lunate fossa and radius are in a good position and the capitate head is intact [28].

Prognosis and outcomes 

The main goal in the management of KD varies depending on the disease stage. In early stages, the aim is revascularisation of the lunate, and in later, more advanced stages, it is symptomatic management, pain control, and preservation of hand function [20].

In most studies, age is identified to be a leading factor affecting prognosis. Adolescent patients, often referred to as “Teenbock” patients, and elderly patients were found to have a better prognosis in comparison to other age groups [14]. Some studies show a better prognosis and self-limiting with a return to normal or near-normal outcome in paediatric patients in the infantile and early juvenile age (13-14 years). However, the same cannot be said for those older than 15 years old presenting with advanced stages of KD, as they are frequently found to have unsuccessful conservative management and often require surgical management [26].

There is no consensus on the exact cause of KD; however, it is widely agreed that anatomic factors cause the lunate to be at risk, thus predisposing patients to it [18].

Limitations 

Upon reviewing existing literature, the majority of focus is on the staging and how it coincides with treatment modalities and the different treatment options available. Unfortunately, the clinical presentation of KD, any red flag symptoms, or literature looking at evidence of disease progression clinically is lacking. The pathogenesis of KD remains poorly understood, and the literature is scarce.

In this narrative review, I recommend the development of a national or international multi-centre cohort study to better determine the prevalence of KD and to provide a more detailed description of its presenting signs and symptoms.

## Conclusions

Avascular necrosis of the lunate, which is known as KD, mostly affects adults between 20 and 40 years of age, males more than females, and high chance in laborers with repetitive trauma or strain on their carpal bones. KD remains to have low prevalence generally and is not yet widely understood or published about in terms of pathophysiology or signs and symptoms. The exact method in which it progresses remains unknown, with studies within the past 5-10 years mostly focusing on surgical treatment options rather than disease progression. The majority of existing research focuses on the treatment modalities, despite the absence of consensus on the appropriate surgical treatment. In addition to this, limited literature exists about the clinical presentation of KD, and it can often be missed in the early stages due to this.
